# Aqueous and Alcoholic Extracts of Triphala and Their Active Compounds Chebulagic Acid and Chebulinic Acid Prevented Epithelial to Mesenchymal Transition in Retinal Pigment Epithelial Cells, by Inhibiting SMAD-3 Phosphorylation

**DOI:** 10.1371/journal.pone.0120512

**Published:** 2015-03-20

**Authors:** Shanmuganathan Sivasankar, Ramu Lavanya, Pemaiah Brindha, Narayanasamy Angayarkanni

**Affiliations:** 1 R.S. Mehta Jain Department of Biochemistry and Cell Biology, Kamalnayan Bajaj Institute for Research in Vision and Ophthalmology, Vision Research Foundation, Chennai, India; 2 School of Chemical and Biotechnology, SASTRA University, Thanjavur, India; 3 Centre for Advance Research in Indian system of Medicine, SASTRA University, Thanjavur, India; Medical University of South Carolina, UNITED STATES

## Abstract

Epithelial to Mesenchymal Transition (EMT) of the retinal pigment epithelium is involved in the pathogenesis of proliferative vitreoretinopathy (PVR) that often leads to retinal detachment. In this study, Triphala, an ayurvedic formulation and two of its active ingredients, namely chebulagic acid and chebulinic acid were evaluated for anti-EMT properties based on *in vitro* experiments in human retinal pigment epithelial cell line (ARPE-19) under TGFβ1 induced conditions. ARPE-19 cells were treated with TGFβ1 alone or co-treated with various concentrations of aqueous extract (AqE) (30 - 300 μg/ml); alcoholic extract (AlE) (50 - 500 μg/ml) of triphala and the active principles chebulagic acid (CA) and chebulinic acid (CI) (CA,CI: 50 - 200 μM). The expression of EMT markers namely MMP-2, αSMA, vimentin and the tight junction protein ZO-1 were evaluated by qPCR, western blot and immunofluorescence. The functional implications of EMT, namely migration and proliferation of cells were assessed by proliferation assay, scratch assay and transwell migration assay. AqE, AlE, CA and CI reduced the expression and activity of MMP-2 at an ED50 value of 100 μg/ml, 50 μg/ml, 100 μM and 100 μM, respectively. At these concentrations, a significant down-regulation of the expression of αSMA, vimentin and up-regulation of the expression of ZO-1 altered by TGFβ1 were observed. These concentrations also inhibited proliferation and migration of ARPE-19 cells induced by TGFβ1. EMT was found to be induced in ARPE-19 cells, through SMAD-3 phosphorylation and it was inhibited by AqE, AlE, CA and CI. Further studies in experimental animals are required to attribute therapeutic potential of these extracts and their active compounds, as an adjuvant therapy in the disease management of PVR.

## Introduction

Proliferative Vitreoretinopathy (PVR) is one of the complicated disorders of the eye and a leading cause for the failure of retinal reattachment surgery in rhegmatogenous retinal detachment (RRD) patients. Retinal detachments can occur due to ocular trauma, high myopia, psuedophakia, aphakia as well as idiopathic causes[1–3]. In PVR, there is characteristic membrane formation on the retina, called as the epi-retinal membrane (ERM). It reflects the wound healing response, elicited by retinal detachment (RD), that ends up in fibrosis which further leads to tractional retinal detachment [1,3,4]. ERM consists of extra cellular matrix proteins and various types of cells. Retinal pigment epithelial cells (RPE) are the predominant cells seen, owing to the dispersion of viable RPE into vitreous, during retinal breaks[5–7]. RPE are cuboidal cells that arrange as a monolayer, forming outer blood retinal barrier of the eye. They are mitotically quiescent throughout adult life. However, they undergo proliferation, migration and form ERM during PVR [2,4]. RPE undergo Epithelial to Mesenchymal transition (EMT), a process that underlies the pathology of PVR [8]. EMT refers to cells having mesenchymal phenotype generated from epithelial cells, as a programmed event, during embryogenesis [9,10]. In pathological organ fibrosis and in cancer metastasis, EMT is however mediated by inflammation and epigenetic changes [9]. Growth factors that accumulate in the vitreous of PVR patients may induce EMT. The cells in the PVR membranes secrete, as well as respond to the growth factors that accumulate in the vitreous [11,12]. *In vitro* studies also show that RPE undergo mesenchymal transition upon TGFβ1 induction [13,14].

In addition to surgical intervention for retinal detachment, anti-proliferative agents and anti-inflammatory agents have been tried as adjuvant therapy in PVR, to prevent proliferation and migration of RPE cells. However, they showed mixed responses in clinical experiments [1,4]. New molecules and molecular targets are therefore warranted for the disease management of PVR. Triphala, commonly used in the Indian ayurvedic medicine, is a combination of the dried fruit powder of three different plants namely *Terminalia chebula*, *Terminalia belerica and Emblica officinalis*. Chebulagic acid (CA) and chebulinic acid (CI) are known to be present in triphala as major constituents [15]. These compounds are reported to have anti-cancer and anti-proliferative properties [16–20]. However, there are no reports on their effects on EMT. This study investigates, if AqE, AlE of triphala and two of its active compounds, CA and CI possess anti-EMT property based on the *in vitro* study in ARPE-19 cells.

## Materials and Methods

### Preparation of Aqueous Extract of Triphala (AqE)

30 g of Triphala churna (mixture of the dried powder of T*erminalia chebula*, *Terminalia belerica and Emblica officinalis*, in the ratio of 1:1:1), (IMPCOPS, Chennai, India) was boiled in 100 ml of Milli-Q water, till the volume was reduced to 1/3^rd^ and was filtered through Whatmann filter paper (Sigma-Aldrich, St.Louis, MO, USA). The filtrate was centrifuged at room temperature to remove debris. The supernatant was lyophilized to get the powdered aqueous extract of triphala (AqE). AqE dissolved in PBS at a concentration of 3 mg/ml was filter sterilized using 0.22 μm membrane filter (Millipore Massachusetts USA), before treating the cells.

### Preparation of Alcoholic Extract of Triphala (AlE)

50 g of Triphala churna was extracted in 150 ml of ethanol using Soxhlet apparatus. The extract was evaporated in vacuum and dried at 40°C to get the alcoholic extract of Triphala (AlE). AlE, dissolved in PBS containing 0.1% DMSO at a concentration of 3 mg/ml was filter sterilized before treating the cells.

### Chebulagic acid (CA), chebulinic acid (CI) and Gallic acid (GA) preparation

CA, CI were purchased from Natural Remedies Pvt. Ltd, Bangalore, India. GA was from Sigma-Aldrich. CA, GA or CI were dissolved in PBS (2 mg/ml) and filter sterilized before treating the cells.

### High Performance Thin Layer Chromatography analysis

For high performance thin layer chromatography (HPTLC), aluminium plates pre-coated with silica gel 60 F 254 (10 × 10 cm) of 0.2 mm thickness (E. Merck, Germany) were used as the stationary phase. The extracts of triphala and the standards (CA, CI and GA (1 mg/ml)) were spotted on silica gel using automatic TLC applicator Linomat V. The composition of the mobile phase used was toluene: ethyl acetate: formic acid (2:5:1.5) (Ayurvedic Pharmacopia). The optimized chamber saturation time for the mobile phase was 30 min at room temperature. The plate was developed in the solvent system, using twin trough chamber and allowed to dry at room temperature. The plates were scanned at A_254_ nm. The images were captured on CAMAG REPROSTAR 3 with win-CATS software.

### Cell culture and Treatment

Adult human Retinal Pigment Epithelial cells, ARPE-19 (ATCC-CRL-2302) was cultured using DMEM-F12 (Sigma-Aldrich) supplemented with 10% (v/v) fetal bovine serum (Gibco, Life Technologies, Carlsbad, CA, USA), 14.2 mM Sodium bicarbonate and 1X solution of Antibiotic-Antimycotic (Gibco).

#### Culturing and treatment of Bovine RPE cells (BRPE)

Bovine eyes were procured from slaughter house. The anterior segments, vitreous and the retina were removed carefully. DMEM-F12 media was added to the eye cup and RPE was gently released using sterile cotton swab. The media was aspirated and the cells were pelleted by centrifugation at 1500 *g* for 10 min. The pellet was plated in T-25 flask containing DMEM-F12 +10% FBS. Cells upto passage numbers 4 were used.

#### Experimental protocol

ARPE-19 cells / BRPE cells (5 x 10^4^) were seeded to 12-well plates and grown to 80% confluence. The cells were serum starved overnight in DMEM-F12 + 1% FBS, followed by the treatment with TGFβ1 (5 ng/ml) (Abcam, Cambridge, UK), for 36 h and 48 h to induce MMP-2 secretion and EMT changes. Varying concentrations of AqE (30–300 μg/ml), AlE (10–500 μg/ml), CA (50–200 μM) or CI (50–200 μM) were given as co-treatment with TGFβ1 to evaluate the anti-EMT properties of the triphala extracts and the active principles. TGFβ1 receptor inhibitor, LY364947 (12.5 μM) and SMAD-3 phosphorylation inhibitor, SIS3 (25 μM) were administered for 6 h, before the addition of TGFβ1 to probe the role of canonical SMAD-3 signalling in EMT on ARPE-19 cells. At the end of 36 h, mRNA expression of EMT markers and MMP-2 at activity and protein level were assessed by qPCR, zymography and ELISA respectively. At the end of 48 h, EMT markers were assessed at protein level by western blot and immunofluorescence. SMAD-3 phosphorylation was assessed between 15 min to one hour of TGFβ1 induction. Western blot analysis showed a maximal phosphoryation at 30 minutes. Hence we assessed the effect of AqE, ALE, CA and CI on SMAD-3 phosphorylation after 30 min of treatment.

#### Cytotoxicity assay

MTT (3-(4, 5-dimethylthiazolyl-2)-2, 5-diphenyltetrazolium bromide) assay was done to check the cytotoxic effect of AqE, AlE, CA, CI and GA in ARPE-19 cells. ARPE-19 cells (5000 cells/well) were seeded in 96-well plate, grown to 80% confluence and serum starved overnight. Various concentrations of AqE (30–300 μg/ml), AlE (50–500 μg/ml), CA (50–200 μM), CI (50–200 μM) or GA (50–300μM) was added to the wells and incubated for 48 h. After incubation, the conditioned medium was replaced with fresh DMEM-F12+1% FBS followed by addition of 20 μL of MTT (to a final concentration of 1 mg/ml). After incubation at 37°C for 3.5 h, in the CO_2_ incubator, the medium was carefully aspirated and formazon crystals formed were dissolved in DMSO. The absorbance was measured at A_570_ nm, with the reference wavelength at A_650_ nm, using an ELISA plate reader. (Spectromax M2E, Molecular Devices, California)

#### Crystal violet dye exclusion assay for cell proliferation

Crystal violet dye which stains the nucleus was used to evaluate cell proliferation as per the method of Rothmier *et al*. [21], with minor modifications. Briefly, 1 x 10^4^ ARPE-19 cells were seeded in 24-well plates, allowed to attach and serum starved overnight. The cells were treated with TGFβ1 alone and co-treated with varying concentrations of AqE (30 and 100 μg/ml), AlE (10 and 50 μg/ml), CA or CI (10 and 100 μM) for 48 h. At the end of the treatment, cells were washed with PBS, fixed with 4% paraformaldehyde for 10 min. The nucleus was stained with 0.5% crystal violet in 20% methanol and the unbound crystal violet was removed by washing it thrice with distilled water. Cells were air-dried and the crystal violet in the cells was dissolved in 0.1 M sodium citrate in 50% ethanol. The absorbance of crystal violet was measured at A_540_ nm and at A_670_ nm as secondary wavelength.

#### Zymography for the activity assay of MMP-2

Cell-free conditioned medium was collected for zymography, after 36 h of exposure. Protein concentration of the conditioned medium was estimated using Bradford’s reagent (Thermoscientific). Thirty five microgram protein was loaded and resolved in 10% polyacrylamide gel containing 1.5% gelatin substrate. The gels were washed in 2.5% Triton-X 100 for 30 min and in Milli-Q water thrice at an interval of 10 min, followed by incubation in developing buffer (50 mM Tris-HCL pH 7.5 containing 200 mM NaCl, 10 mM CaCl_2_, 0.05% Brij-35 and 0.02% Sodium azide) for 18 h at 37°C. Gels were stained using Coomassie brilliant blue-R 250 solution containing 40% methanol and 10% acetic acid. A mixture of 40% methanol and 10% acetic acid without Coomassie-R 250 was used for destaining the gel. The gelatinolytic activity was visualized as a zone of clearance against a blue background. Gels were scanned using GS800-calibrated densitometer (BioRad, Hercules, CA USA).

#### MMP-2 estimation by Enzyme Linked Immunosorbent assay

MMP-2 level in the conditioned medium was quantified using Quantikine ELISA kit (R&D systems, Minneapolis, USA) as per the manufacturer’s guidelines.

#### RNA extraction and Polymerase chain reaction

Total RNA was extracted using TRIzol reagent (Life Technologies) as per manufacturer’s instruction, and it was quantified by Nanodrop spectrophotometer. One microgram of total RNA was converted to cDNA using iScript cDNA synthesis kit (Bio-Rad). Quantitative PCR (qPCR) based on SYBR green Chemistry was done using Roche Light Cycler-96 PCR system (Roche, Basel, Switzerland). qPCR primers were designed as per Basu and Thornton [22] and the sequences are given in Table 1. Specificity of the amplified product was analyzed using corresponding melting curve. For calculating fold change, comparative 2^ΔCt (treated—untreated)^ method was used. [23].

**Table 1:** List of Primers used for PCR.

S.No	Gene	Forward Primer	Reverse Primer	Reference
1	18s rRNA	5’- GTGGAGCGATTTGTCTGGTT-3’	5’- GGACATCTAAGGGCATCACAGA-3’	[24]
2	MMP-2	5’-CAGGAGGAGAAGGCTGTGTT-3’	5’-TTAAAGGCGGCATCCACTCG-3’	
3	αSMA	5’-GGCTGTTTTCCCATCCATTGT-3’	5’-TCTTTTGCTCTGTGCTTCGT-3’	
4	Vimentin	5’-TTGCAGGAGGAGATGCTTCA-3’	5’-TTGCAGGAGGAGATGCTTCA-3’	
5	ZO-1	5’-AGTAAGTCGTCCTGATCCTGAACC-3’	5’-TCCTTCTGTTAACCACACCACTCC-3’	

#### Scratch Assay for migration

ARPE-19 cells (6 x 10^4^) were seeded in 6-well plates grown to 80% confluence and serum starved overnight. A scratch was made in each well using a sterile 200 μl pipette tip and washed with PBS to remove floating cells. Cells were treated with TGFβ1 (5 ng/ml) alone or co-treated with AqE (100 μg/ml), AlE (50 μg/ml), CA (100 μM) or CI (100 μM). Untreated cells were used as control. Migration of cells towards wound closure was monitored and images were captured at 36 h using a phase contrast microscope (EVOS-XL-core, American Microscopy group, Life Technologies).

#### Transwell migration assay

After treatment with TGFβ1 and co-treatment with the extracts and the active compounds, the cell-free conditioned medium were collected and used for migration assay. The Transwell migration assay was performed as described by Eischler *et al*. accordingly, millicell inserts (Millipore) of 8 μm pore size, coated with 1 mg/ml gelatin on both sides were used. ARPE-19 cells (2.5 x 10^4^) were seeded and the conditioned medium was added to the upper chamber, while the lower chamber had DMEM-F12+10% FBS. After 18 h, the cells were permeabilized, fixed with methanol and stained with Giemsa. Non-migrated cells were scraped off from the upper surface and the migrated cells in the lower surface were observed under phase contrast microscope at 40X magnification. Five random fields were captured for a single insert and the average of the number of migrated cells was counted. Three independent experiments were done and the cell number expressed as mean ± SD.

#### Immunocytochemistry

ARPE-19 cells (2.5 x 10^4^) were grown to 80% confluence in 4-well chamber slides (Nunc NALGENE, New York, USA) and serum starved overnight. Cells were treated with TGFβ1 (5 ng/ml) or co-treated with AqE (100 and 300 μg/ml) or AlE (50 and 500 μg/ml) or CA or CI (100 μM) for 48 h. DMSO (0.1%) was used as a vehicle control. After treatment, the cells were washed with PBS, fixed with 4% paraformaldehyde, permeabilized using 0.1% Triton-X100 for 10 min and blocked with 2% BSA for 60 min. After overnight incubation with primary antibody of αSMA (1:100) (Sigma-Aldrich) or vimentin (1:100) (Abcam) or ZO-1 (1:75) (Abcam), the chambers were rinsed with PBS, followed by incubation with appropriate secondary antibody (1:400) for 2 h. Unbound secondary antibody was removed by washing with PBST. The nucleus was stained with DAPI. The slides were air-dried and mounted using 1:1 mix of glycerol and PBS. All the images were captured using a fluorescence microscope (Carl-Zeiss). Experiments were repeated thrice and 5 random images were captured for a single chamber in the slide.

#### Western blot analysis

ARPE-19 cells were lysed in RIPA buffer (50 mM Tris-HCL, 150 mM NaCl, 0.1% Triton-X 100, 0.5% sodium deoxycholate, 0.1% SDS and protease inhibitor cocktail (Sigma-Aldrich)). For experiments pertaining to analysis of SMAD-3, M-Per protein extraction reagent (Thermoscientific) containing protease and phosphatase inhibitor cocktail (Sigma-Aldrich) was used. The total protein was estimated by BCA assay (Thermoscientific) and 40 μg of protein was resolved in 10% SDS PAGE, and the blot was transferred to PVDF membrane (Hybond Amersham). Four percent of non-fat dry milk or 4% BSA (for phosphorylated SMAD-3) in TBST was used for blocking. Blots were incubated overnight with appropriate dilution of primary antibody for N-Cadherin (1:4000, abcam), αSMA (1:4000, Sigma-Aldrich), vimentin (1:4000, abcam), ZO-1 (1:1000, invitrogen), total SMAD-3 (1:5000, CST), phosphorylated SMAD-3 (1:4000, CST) and βactin (1:8000 Santa Cruz biotech). After washing unbound primary antibody, the membranes were incubated for 2 h with appropriate secondary antibody, i.e., anti-mouse (1:40,000, Santa Cruz biotech) or anti-rabbit (1:40,000, Santa Cruz biotech). Bands were detected using ECL prime reagent (GE Amersham) and the image was captured using Flourchem FC3 Gel documentation system. Intensity of bands was assessed using Image-J software. βactin was used as loading control. p-SMAD-3 / SMAD-3 ratio normalized to βactin was used to measure the phosphorylation status of SMAD-3.

### Statistics

The data are expressed as Mean ± SD, of three independent experiments done in triplicates. ANOVA with Bonferroni correction was done to calculate significance. P value, <0.05 was considered significant.

## Results

### Chebulagic Acid (CA), Chebulinic Acid (CI) and Gallic acid (GA) are present in the aqueous extract of triphala (AqE) and alcoholic extract of triphala (AlE)

**Fig 1:**
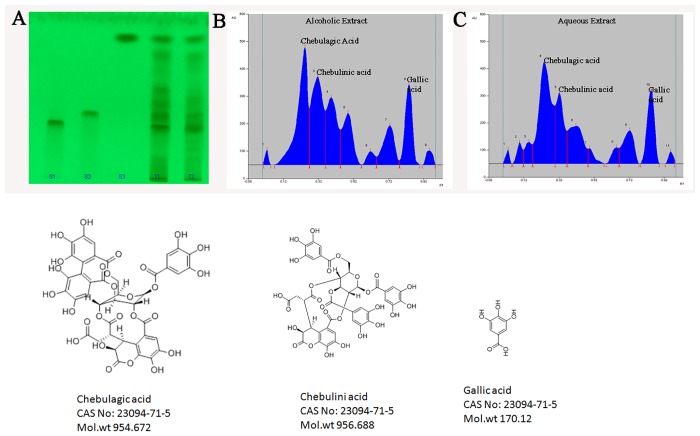
HPTLC analysis of aqueous extract of Triphala (AqE) and alcoholic extract of Triphala (AlE). (A) HPTLC of Standards: Chebulagic acid (S1), Chebulinic acid (S2), Gallic acid (S3), AlE (T1), AqE (T2). (B) and (C) are chromatograms of AlE and AqE respectively.

HPTLC was done to validate the extraction of triphala. CA, CI and GA were present in both aqueous and alcoholic extracts of triphala (Fig. 1). Standard chromatogram in HPTLC showed the Rf value of CA as 0.25, CI as 0.32 and GA as 0.84 (Fig. 1A). Peaks detected in the AqE (4, 5 and 10) and AlE (2, 3 and 8) show the presence of CA, CI and GA in the extracts of triphala (Fig. 1B, C).

### AqE, AlE, CA and CI are non-cytotoxic to the ARPE-19 cells

**Fig 2:**
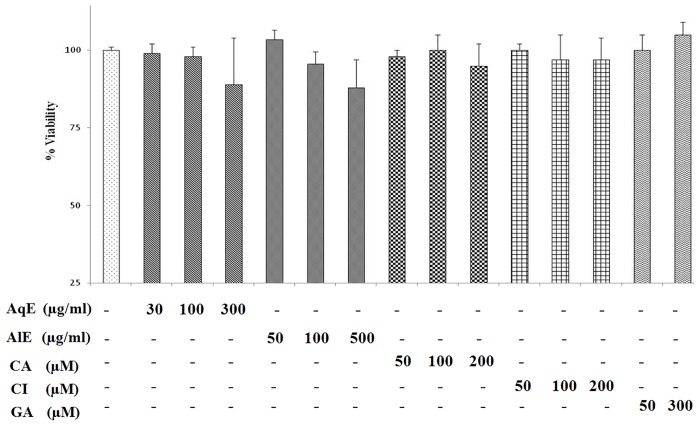
Cytotoxicity assay for AqE, AlE, CA and CI in ARPE-19 cells. MTT assay showing the effect of AqE, AlE, CA, CI and GA on viability of ARPE-19 cells. Data represents Mean ± SD of three independent experiments in triplicates.

MTT assay was done to check for the cytotoxic effect of the compounds studied in ARPE-19 cells. Cells were treated with AqE (30–300 μg/ml), AlE (50–500 μg/ml), CA and CI (50–200 μM) or GA (50 and 300 μM) for 48 h. Viable cell percentage was calculated based on absorbance of formazon crystals formed (Fig. 2). AqE (300 μg/ml) and AlE (500 μg/ml) showed 89% and 88% cell viability. Viability was more than or equal to 95%, in all other concentrations of AqE and ALE. CA, CI and GA showed no toxicity, as the viability was more than 95% in the tested concentration.

### AqE, AlE, CA and CI inhibited MMP-2 activity and protein expression in ARPE-19 cells

**Fig 3:**
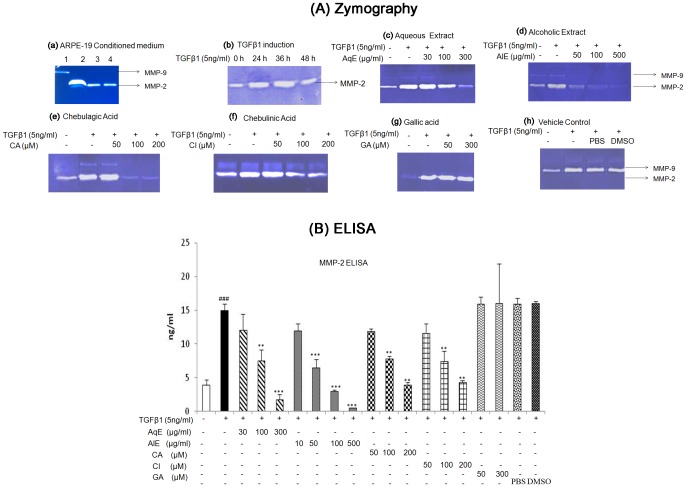
AqE, AlE, CA and CI reduced MMP-2 induced by TGFβ1. Treatment of ARPE-19 cells with TGFβ1 alone or co-treated with various concentrations of AqE, AlE, CA, CI or GA for 36 h. The cell free conditioned media was used for gelatine zymography (A). Untreated ARPE-19 cells showing secretion of MMP-2 (Lane 1: MMP-9 standard, 2: MMP-2 standard, 3,4: ARPE-19 conditioned medium) (a); TGFβ1 (5 ng/ml) inducing MMP-2 activity at 36 h (b); Effect of AqE (c), AlE (d), CA (e), CI (f), GA (g) and vehicle control (h) on MMP-2 activity by zymography. Quantitation of MMP-2 levels by using ELISA (B). Data represents Mean ± SD of at least three independent experiments in triplicates. p value: ^###^p<0.001 is the comparison between control vs. TGFβ1 and p values: **p<0.01, ***p<0.001are comparison between TGFβ1 and corresponding treatment.

ARPE-19 cells secreted MMP-2 into the medium (Fig. 3A: a) which was further induced by TGFβ1 (5 ng/ml) at 36h (Fig. 3A: b). Treatment with AqE, AlE, CA and CI, decreased TGFβ1-induced MMP-2 activity (Fig. 3A: c-f). But gallic acid did not reduce MMP-2 activity (Fig. 3A: g). TGFβ1 treatment increased the MMP-2 protein level significantly to 14.9 ± 0.9 ng/ml compared to untreated control 3.8 ± 0.8 ng/ml. Treatment with AqE, AlE, CA and CI down-regulated the MMP-2 protein levels dose-dependently and significantly. GA did not have an effect on MMP-2 levels induced by TGFβ1 (Fig. 3B). The ED 50 value for MMP-2 inhibition was found to be 100 μg/ml for AqE, 50 μg/ml for AlE and 100 μM for both CA and CI.

### AqE, AlE, CA and CI inhibited expression of EMT markers

**Fig 4:**
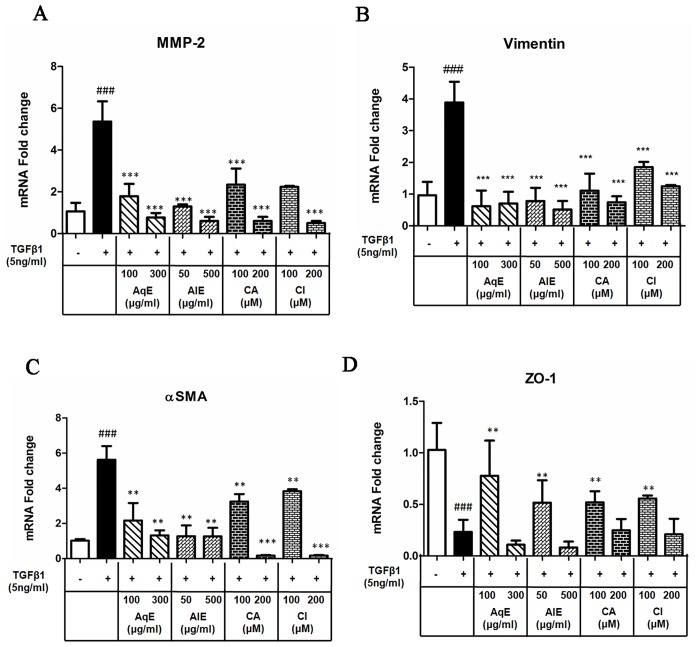
Effect of AqE, AlE, CA and CI on mRNA expression of EMT markers. Quantitative real time PCR showing the mRNA expression of MMP-2 (A), vimentin (B), αSMA (C) and ZO-1 (D) in cells treated with TGFβ1 alone and co-treated with AqE, AlE, CA or CI for 36 h. Data represents Mean ± SD of three independent experiments in triplicates. p values:^###^p<0.001 is the comparison between control vs. TGFβ1 and p values: *p<0.05, **p<0.01, ***p<0.001 are comparisons between TGFβ1 and corresponding treatment.

ARPE-19 cells were treated with TGFβ1 alone or in combination with AqE, AlE, CA and CI for 36 h and the mRNA expression of mesenchymal markers (MMP-2, αSMA, vimentin) and epithelial marker ZO-1 was assessed by qPCR. The qPCR results showed that the TGFβ1 treatment increased the mRNA expression of MMP-2, vimentin and αSMA, which was significantly down-regulated by co-treatment with AqE, AlE, CA and CI (Fig. 4A-C). While TGFβ1 decreased the ZO-1 expression, co-treatment with AqE (100 μg/ml), AlE (50 μg/ml), CA and CI (both at 100 μM) improved the expression of ZO-1. However, at higher concentrations, this was not observed (Fig. 4D).

**Fig 5:**
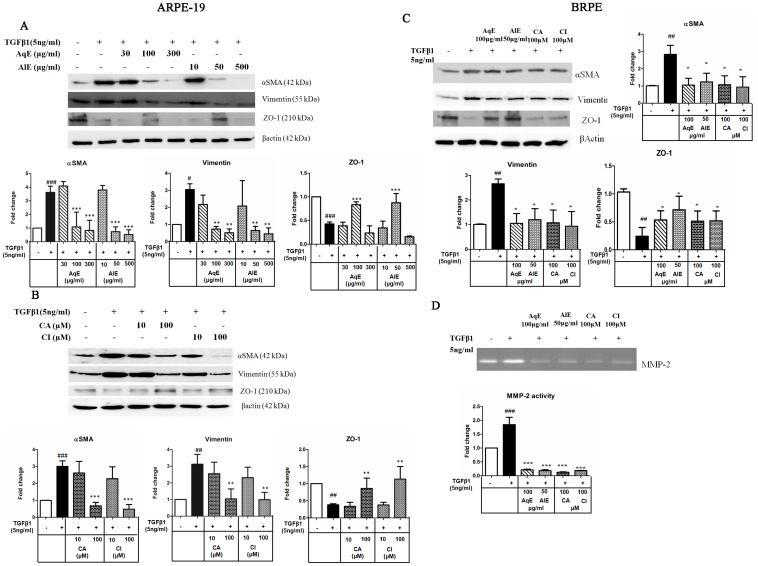
Effect of AqE, AlE, CA and CI on EMT markers in ARPE-19 and BRPE cells. Western blot showing the effect of AqE (30–300μg/ml), AlE (10–500 μg/ml) (A); CA and CI (10–100 μM) (B) on the expression of EMT markers, altered by TGFβ1 in ARPE-19 cells. Western blot (C) and zymography (D) showing the effect of AqE (100 μg/ml), AlE (50 μg/ml), CA (100 μM) and CI (100 μM) on EMT markers, altered by TGFβ1 in BRPE cells. Band intensity was quantified using NIH image-J software and expressed as fold change after normalization with βactin. Data represents Mean ± SD of three independent experiments. p values:^#^p<0.05, ^##^p<0.01, ^###^p<0.001 are comparisons between control and TGFβ1. p values: **p<0.01, ***p<0.001 between TGFβ1 and corresponding treatment.

**Fig 6:**
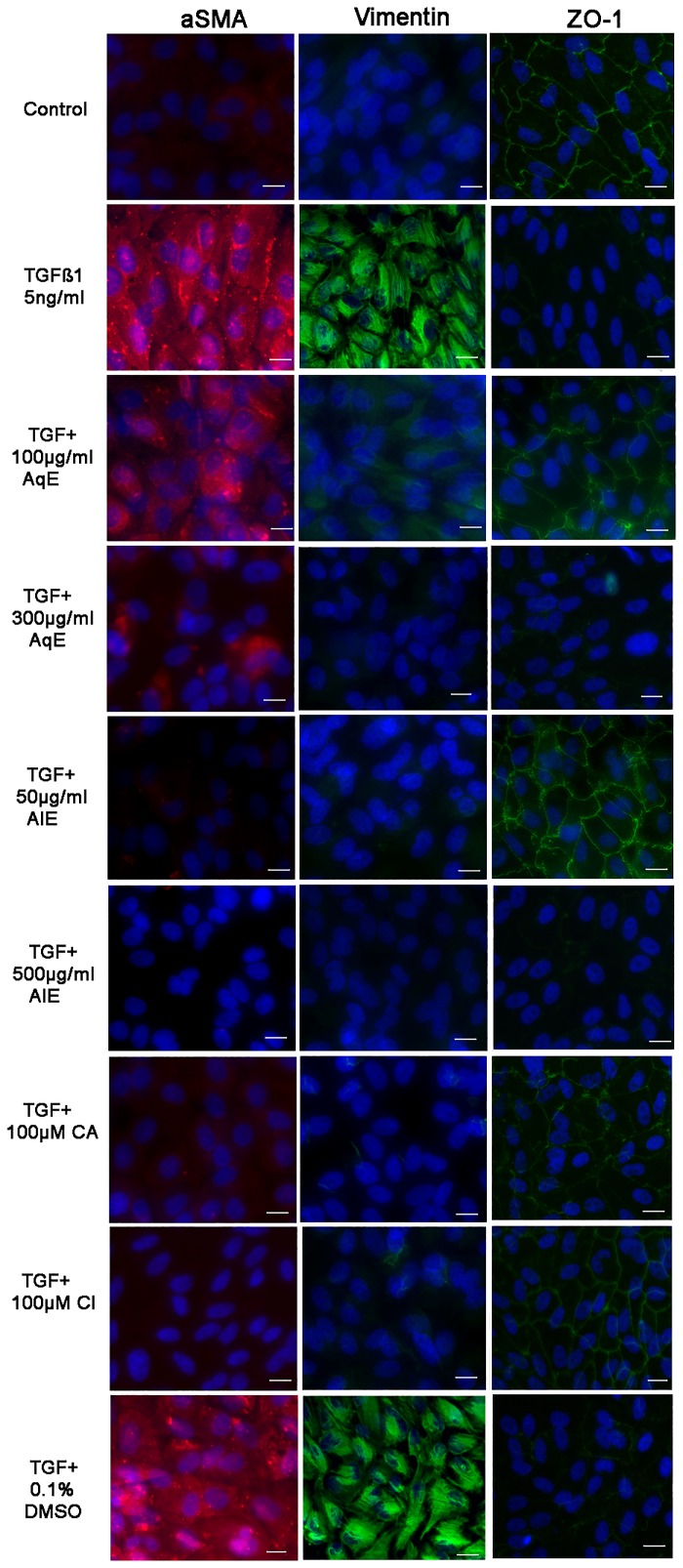
Effect of AqE, AlE, CA and CI on protein expression of αSMA, Vimentin and ZO-1 by Immunofluorescence. ARPE-19 cells were seeded in chamber slides, treated with TGFβ1 or co-treated with TGFβ1 and various concentrations of AqE, AlE, CA and CI as indicated for 48 h. Cells were immunostained for αSMA (Red fluorescence), vimentin and ZO-1 (Green fluorescence). Image magnification 40X, Scale bar 10μm.

Western blot analysis also revealed that TGFβ1 induced the expression of αSMA, vimentin and down-regulated the ZO-1 expression. AqE and AlE decreased the mesenchymal markers, dose-dependently. However, only at 100 μg/ml of AqE and 50 μg/ml of AlE, ZO-1 expression showed an increase (Fig. 5A). CA and CI also showed a dose-dependent decrease in the expression of mesenchymal markers and up-regulated the epithelial marker ZO-1 (Fig. 5B). In BRPE cells, TGFβ1 induced EMT, as seen by decreased ZO-1 expression and increased expression of αSMA and vimentin. Treatment with AqE (100 μg/ml), AlE (50 μg/ml), CA (100 μM) and CI (100 μM), significantly reversed these expressions (Fig. 5C). Similarly TGFβ1 induced MMP-2 activity, which was also decreased significantly by AqE, AlE, CA and CI. (Fig. 5D). Immunocytochemistry (Fig. 6) also reflects the same pattern of expression as seen in qPCR and western blot.

### AqE, AlE, CA and CI prevented the proliferation and migration of ARPE-19 cells

**Fig 7:**
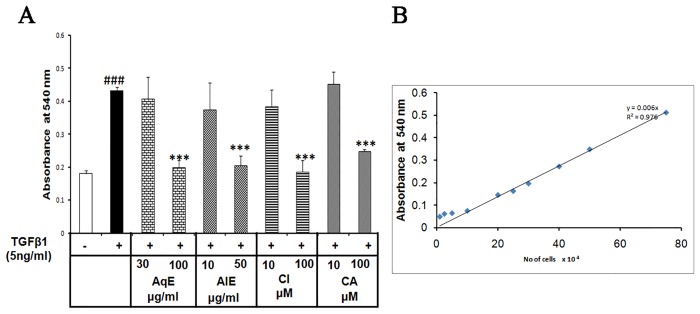
AqE, AlE, CA and CI prevent proliferation of ARPE-19 cells. Crystal violet dye exclusion showing the effect of TGFβ1 treatment and co-treatment with AqE, AlE, CA or CI on cell proliferation (A). Linear relationship between cell number and absorbance is shown (B). Data represents mean ± SD of three independent experiments done in quadruplicates. p value: ^###^p<0.001 is a comparison between control vs. TGFβ1 treatment. p value: ***p<0.001 is a comparison between TGFβ1 and corresponding treatment.

**Fig 8:**
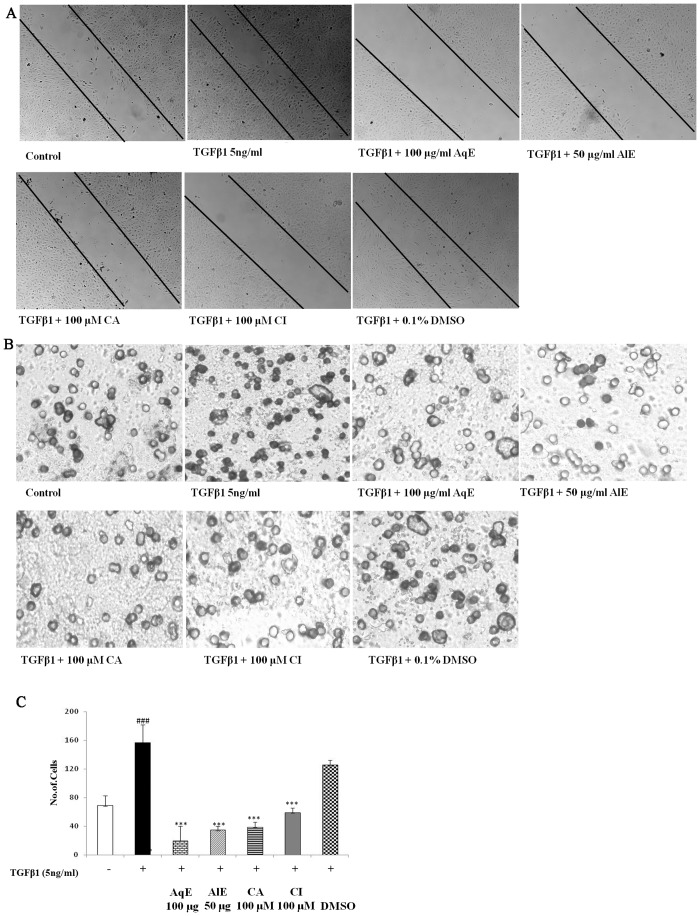
AqE, AlE, CA and CI prevent migration of ARPE-19 cells. Scratch assay showing migratory capacity of ARPE-19 cells upon TGFβ1 induction and on treatment with AqE, AlE, CA and CI (A). The Transwell migration assay showing the effect of TGFβ1 and co-treatment with AqE, AlE, CA and CI on migration of ARPE-19 cells (B). Enumeration of cell number is given in graph (C). All experiments were done thrice independently in triplicates. p value: ^###^p<0.001 is a comparison between control vs. TGFβ1 treatment. p value: ***p<0.001 is a comparison between TGFβ1 and corresponding treatment.

TGFβ1 induced the proliferation of ARPE-19 cells by 2.4 fold as seen by crystal violet assay. Treatment with AqE, AlE, CA and CI reduced the proliferation dose-dependently and significantly (Fig. 7). Scratch assay showed that TGFβ1 induced the migratory capacity of ARPE-19 cells during EMT, while the compounds studied inhibited the same. Cells were observed under microscope from 0 to 36 h periodically and captured at 36 h. TGFβ1 induced the migration of ARPE-19 cells compared to control, which was decreased by AqE, AlE, CA and CI. The vehicle control did not have any effect (Fig. 8A). The Transwell migration assay was done to see the effect of MMP-2 on migration of ARPE-19 cells using conditioned medium collected after treatment protocol. Previous reports showed that MMP-2 can degrade proteins like ZO-1, occludin and enhance migration. The conditioned medium from TGFβ1 treated cells have high MMP-2 activity compared to untreated control. Hence, TGFβ1 treatment is expected to enhance the migration of ARPE-19 cells. In agreement with this, we found that TGFβ1 induced the migration of ARPE-19 significantly by as much as 90%, compared to control. Co-treatment with AqE (100 μg/ml), AlE (50 μg/ml), CA (100 μM) and CI (100 μM) significantly reduced the MMP-2 and in turn, the migration. The vehicle control (DMSO) did not have any effect (Fig. 8B). Treatment with doxycycline, an MMP-2 inhibitor, reduced TGFβ1-induced MMP-2 activity in ARPE-19 cells and prevented migration as seen in the transwell migration assay (S1 Fig).

### TGFβ1 induced EMT through SMAD-3 phosphorylation which was inhibited by AqE, AlE, CA and CI

**Fig 9:**
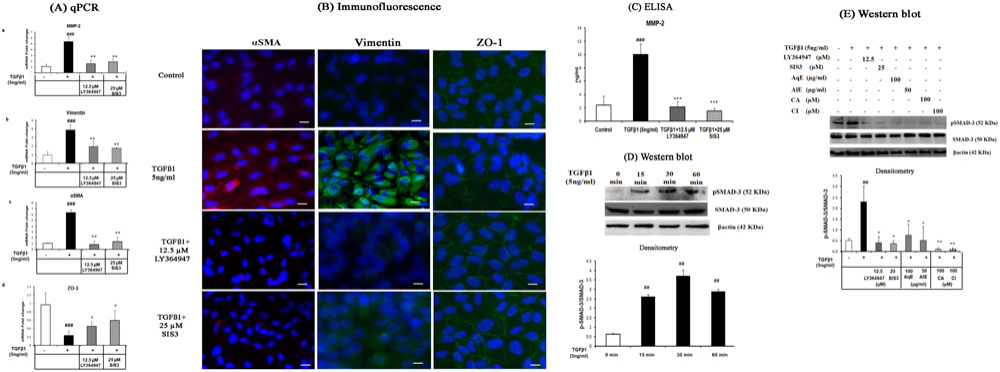
TGFβ1 induces EMT through SMAD-3 phosphorylation, which was prevented by AqE, AlE, CA and CI. Effect of LY364947 and SIS3 on the expression of EMT markers altered by TGFβ1, seen by Quantitative real time PCR (A), Immunofluorescence (B), ELISA (C), Western blot showing TGFβ1 induction of SMAD-3 phosphorylation at various time points (D) effect of AqE, AlE, CA and CI on TGFβ1 induced SMAD-3 phosphorlyation at 30 min (E). Data represents mean ± SD of three independent experiments. p value: ^##^p<0.01 is a comparison between control and TGFβ1 treatment. p values: *p<0.05, **p<0.01 are a comparison between TGFβ1 and corresponding treatment.

TGFβ1 can relay signal through canonical or non-canonical pathway. To see if the canonical pathway was involved, ARPE-19 cells were pre-treated with TGFβ1 receptor inhibitor, LY364947 (12.5 μM) and SMAD-3 specific inhibitor, SIS3 (25 μM) for 6 h, followed by treatment with TGFβ1. mRNA and protein expressions of EMT markers were assessed after 36 h and 48 h of treatment. qPCR showed that LY364947 and SIS3 inhibited TGFβ1-induced EMT, by down-regulating mRNA expression of MMP-2, vimentin and αSMA and up-regulating ZO-1 mRNA (Fig. 9A: a-d). Immunocytochemistry (Fig. 9B) and the MMP-2 levels by ELISA (Fig. 9C) showed a similar observation at the level of protein. LY364947 and SIS3 were screened between 5.0 and 25 μM concentrations. Sixty to eighty percent of inhibition of all the EMT markers was observed at 12.5 μM of LY364947 and 25 μM of SIS3. There was no significant cell death due to these inhibitors at the concentrations studied (Data not shown). Further studies were done to check if the mechanism of EMT inhibition by AqE, AlE, CA and CI occurs through SMAD-3 phosphorylation. TGFβ1 treatment was found to increase phosphorylation of SMAD-3 significantly at 30 min (Fig. 9D) compared to that of control. This was reduced by AqE (100 μg/ml), AlE (50 μg/ml), CA (100 μM) and CI (100 μM) (Fig. 9E). Ly364947 and SIS3 were used as positive controls. Hence, we conclude that AqE, AlE, CA and CI mediate anti-EMT effects by preventing SMAD-3 phosphorylation.

## Discussion and Conclusion

EMT generates mesenchymal-like cells from epithelial cells, and it is characterized by up-regulation of mesenchymal markers like αSMA, MMPs, vimentin, N-Cadherin and down-regulation of epithelial markers like E-Cadherin, ZO-1, cytokeratin [25]. ERM membrane of PVR patients showed positive staining for vimentin [26] and αSMA [27]. The vitreous of PVR patients show elevated levels of MMP-2 [28–30]. *In vitro* experiments have proved that TGFβ1 induces EMT changes [13,14] and MMP-2 secretion in RPE cells [31]. Thus, EMT change has been associated with the pathogenesis of PVR. Hence, inhibition of EMT can be beneficial in the treatment of PVR. This *in vitro* study in human ARPE19 cells reveals that chebulagic acid and chebulinic acid present in triphala extracts inhibit EMT in ARPE-19 cells.

Matrix-Metalloprotease-2 (MMP-2/Gelatinase-A) is a matrix-degrading enzyme that cleaves the basement membrane and helps in the dissemination of cells attached to the extra cellular matrix, thereby promoting migration during EMT [32]. MMP-2 and MMP-9 are reported to degrade the junction proteins such as occludin in ARPE-19 cells [33]. Previously, hyaluronidase and collagenase inhibiting activity of triphala gugulu, another formulation of triphala has been reported [34]. Abraham *et al*., reported on the MMP-9 inhibition of triphala [35]. In this study, we evaluated the anti-MMP-2 activity of the triphala extracts and its active principles CA, CI and GA in APRE19 cells. We found that AqE, AlE, CA and CI down-regulated TGFβ1-induced MMP-2. However, GA did not show any anti-MMP-2 activity as observed by ELISA and zymography. The ED50 value for anti-MMP-2 (both activity and protein) was found to be 100 μg/ml for AqE, 50 μg/ml for AlE, 100 μM for CA and CI. The same concentrations reduced the TGFβ1 induced MMP-2 activity in the primary culture of BRPE cells. This is the first report on the anti-MMP-2 activity of the compounds CA and CI as seen in ARPE-19 and BRPE cells.

Further, the study showed that TGFβ1 induced EMT markers in ARPE-19 and BRPE cells. Treatment with AqE, AlE, CA and CI decreased the expression of vimentin and αSMA in a dose-dependent manner. With respect to ZO-1, the loss incurred due to TGFβ1 treatment was improved by treatment with AqE, AlE, CA and CI, at a relatively lower concentration as used in this study. The cell viability was however not altered at higher concentrations. Such biphasic effects of compounds, based on concentration are reported. Studies point out that the direct effect of polyphenols at higher and lower concentrations have different effects on NFkB and MAPK pathways with different outcomes [36,37]. At higher concentrations of the triphala extracts, the effect of other active ingredients of it might have played an inhibitory role. We observed that gallic acid is one such compound that was found to have no significant inhibitory effect on MMP-2 activity as that of CA or CI, but had adverse effect at a concentration of 600μM, resulting in cell death (Data not shown). In concordance with previous reports [38,39], E-Cadherin mRNA expression was seen in ARPE-19 cells, though no detectable protein expression was observed. Hence, we looked at N-Cadherin expression that was induced by TGF β1, while treatment with AqE, AlE, CA and CI showed a dose–dependent decrease (S2 Fig).

This study is a first report on anti-EMT effect of the triphala extracts as well as its active components, namely CA and CI in ARPE-19 cells. While there is an isolated report on the wound healing property of triphala [40], there are no studies highlighting the anti-EMT activity. However, polyphenols such as Epigallo-catechin-gallate (EGCG) have been shown to exhibit anti-EMT activity by inhibiting vimentin [41].

EMT induces proliferation and migration of RPE cells. AqE, AlE, CA and CI inhibited the proliferation and migration of ARPE-19 cells associated with EMT. Migration is promoted by up-regulated MMP-2. Doxycycline, a known inhibitor of MMP-2 reduced the activity and the migration of ARPE-19 cells. AqE, AlE, CA and CI inhibited the migration of RPE cells through MMP-2 inhibition. Curcumin is reported to inhibit EMT in pancreatic cancer cells [42]. EGCG is shown to prevent migration of RPE cells [43]. Further studies are underway to compare AqE, AlE, CA and CI with other natural compounds like EGCG and curcumin.

TGFβ1 mediates downstream signalling through the tyrosine kinase domain of its trans-membrane receptor, TGFβR1, which phosphorylates SMAD-2 and SMAD-3. Phosphorylated SMAD-2 and SMAD-3 are dimerized and this dimer forms a complex with SMAD-4, which then translocates into nucleus to activate the expression of mesenchymal markers [44,45]. Review by Xu *et al*. 2009 shows that SMAD-3 acts as a master regulator and activates Snail, ZEB and bHLH family of transcription factors for the expression of EMT markers [46]. In this study, ARPE-19 cells treated with TGFβR1 inhibitor, LY364947 and SMAD-3 inhibitor, SIS3 showed inhibition of EMT. SIS3 is a specific inhibitor for SMAD-3 phosphorylation and does not affect the phosphorylation status of p38MAPK or ERK pathway [47]. Hence it is inferred that TGFβ1/TGFβR1/SMAD-3 axis regulates the expression of EMT markers in ARPE-19 cells. This is in agreement with the previous report in the mouse model of PVR, wherein role of p-SMAD-3 in EMT was shown [48]. It also reflects J.West-Mays personal communication, which states that TGFβ1 does not induce EMT in RPE in the absence of SMAD-3 [8]. SMAD-3 mediates EMT in RPE and lens epithelium and inhibition of SMAD-3 phosphorylation can prevent EMT during cataract and PVR [49]. This study reveals that AqE, AlE, CA and CI inhibit EMT and MMP-2 induction in ARPE-19 cells by preventing phosphorylation of SMAD-3, while not affecting the total SMAD-3 levels. Further studies are needed, to see if the compounds in the study additionally influence at the level of TGFβR1, by inhibiting TGFβ binding or by inhibiting phosphorylation activity of RTK domain, or by altering the membrane receptor expression. Moreover, in light of recent reports, EMT is also mediated by Wnt signalling [50,51]. Hence the effect of CA and CI on the Wnt pathway also needs further evaluation.

AqE, AlE, CA and CI inhibited EMT and the EMT mediated migration and proliferation at a concentration of 100 μg/ml, 50 μg/ml, 100 μM and 100 μM, respectively. CA and CI are high molecular weight compounds that exhibit anti-EMT activity at 100 μM concentration in human and in bovine RPE cell, under TGFβ1 induced condition. However, Li *et al*. reported that CI at 2 μM concentration exhibits anti-angiogenic property in HUVEC cells [14]. Thus, the effective concentration seems to depend on the type of cell and the molecular targets as well.

Molecules and strategies such as 5-Flourouracil, daunorubicin (anti-proliferative), alkylphosphocholines, AG1295 (for inhibiting growth factors and signalling), were tried for PVR. But these studies failed to yield significant clinical outcome [4]. Inhibition of EMT can be yet another strategy for PVR management. Our study identified chebulagic acid and chebulinic acid as potent inhibitors of TGFβ1-induced EMT in ARPE-19cells through inhibition of SMAD-3 phosphorylation. These compounds therefore have potential therapeutic value for PVR as adjuvant therapy. Further experimental studies in animal models of PVR are required to validate the same.

## Supporting Information

S1 Fig Effect of Doxycycline on MMP-2 and migration in ARPE-19 cells. Zymography (A) shows that, 25 μg/ml of doxycycline, reduced the MMP-2 activity induced by TGF-β1. Transwell migration Assay (B) shows that doxycycline (25μg/ml), inhibited MMP-2 activity and reduced the migration of ARPE-19 cells induced by TGFβ1.
(TIF)

S2 Fig Effect of AqE, AlE, CA and CI on N-Cadherin expression in ARPE-19 cells. TGFβ1 induced N-Cadherin expression in ARPE-19 cells which is inhibited by AqE and AlE (A), CA and CI (B).
(TIF)
