# Supplementary figures and images for: Aqueous and Alcoholic Extracts of Triphala and Their Active Compounds Chebulagic Acid and Chebulinic Acid Prevented Epithelial to Mesenchymal Transition in Retinal Pigment Epithelial Cells, by Inhibiting SMAD-3 Phosphorylation
Source: PLoS One. 2015 Mar 20;10(3):e0120512. doi: 10.1371/journal.pone.0120512 (PMC4368423; doi:10.1371/journal.pone.0120512)

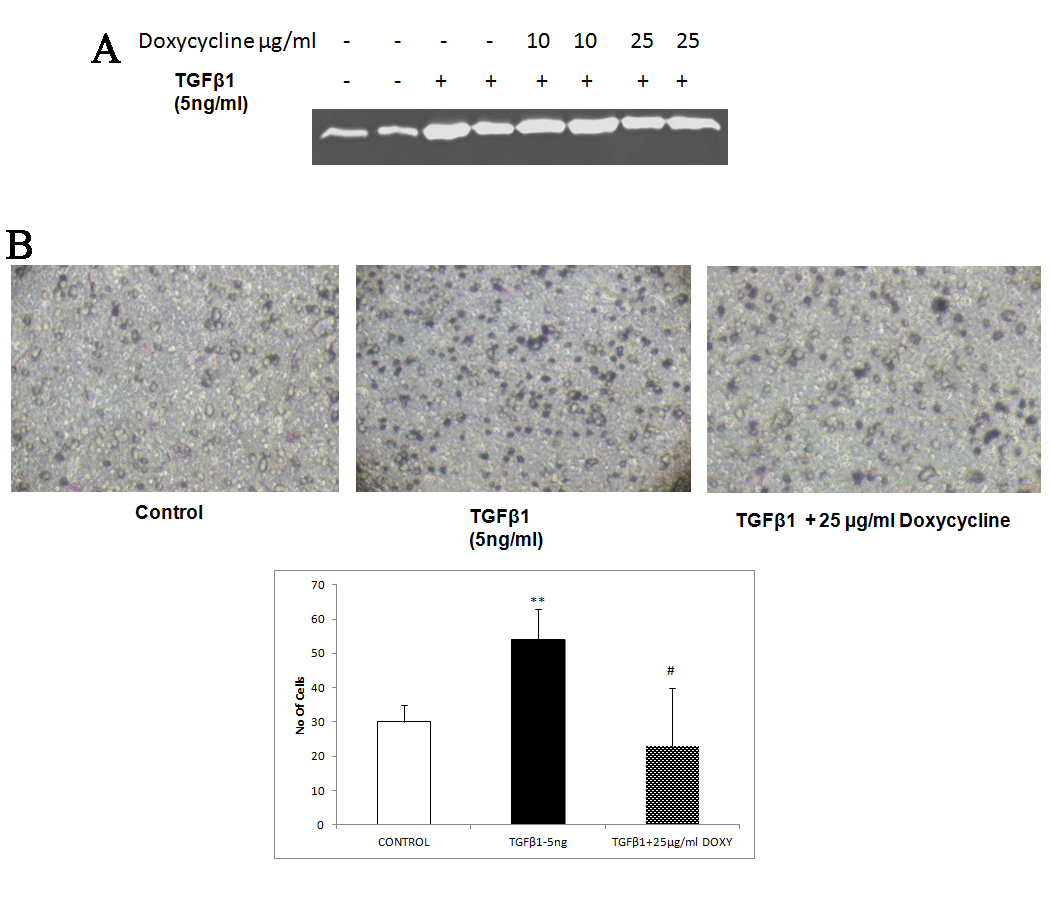

Supplement: S1 Fig — Zymography (A) shows that, 25 μg/ml of doxycycline, reduced the MMP-2 activity induced by TGF-β1. Transwell migration Assay (B) shows that doxycycline (25μg/ml), inhibited MMP-2 activity and reduced the migration of ARPE-19 cells induced by TGFβ1. (TIF) [file pone.0120512.s001.tif]

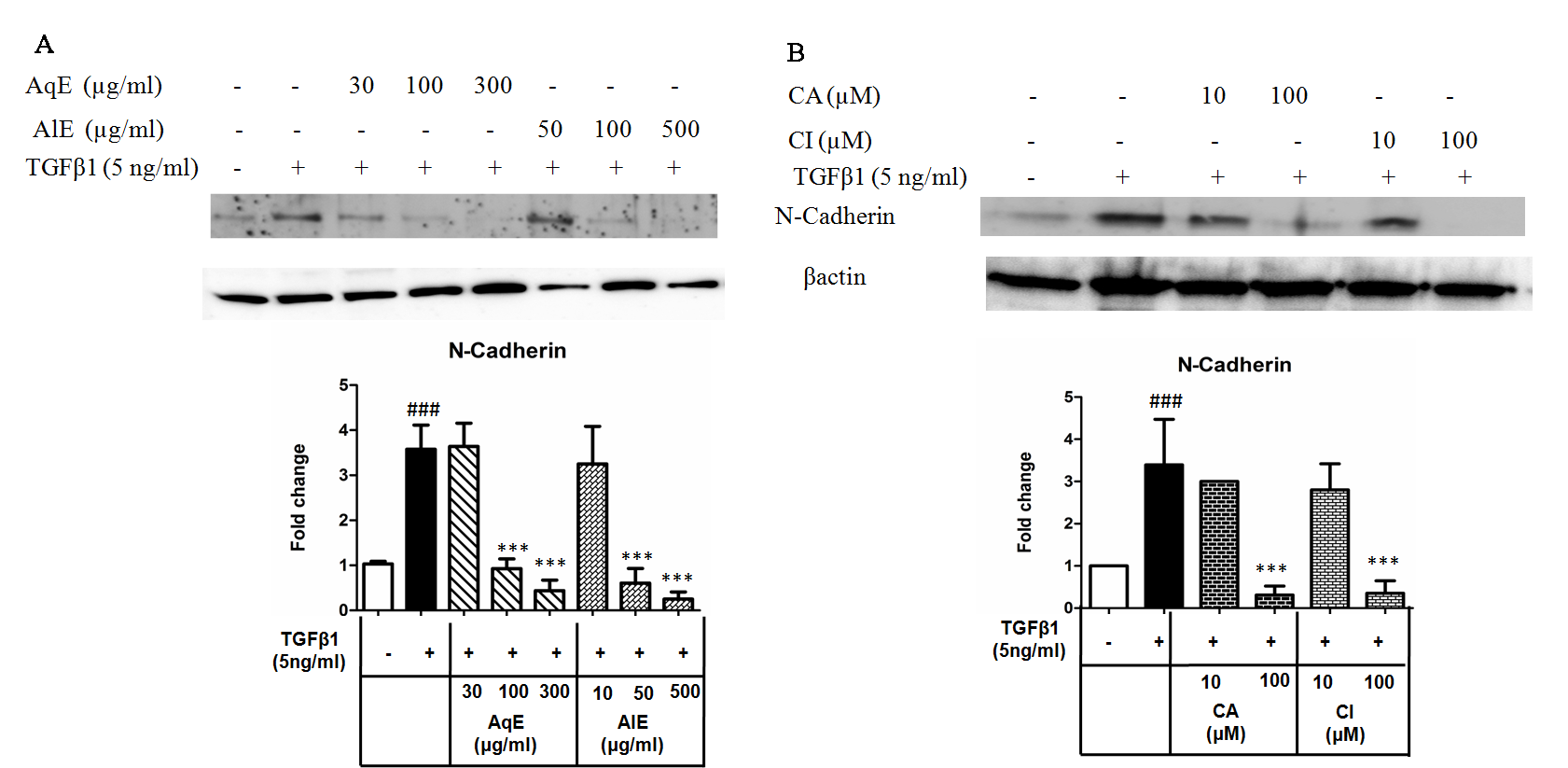

Supplement: S2 Fig — TGFβ1 induced N-Cadherin expression in ARPE-19 cells which is inhibited by AqE and AlE (A), CA and CI (B). (TIF) [file pone.0120512.s002.tif]
